# Communication Challenges and Mitigation Strategies in Primary Care Virtual Consultations: Qualitative Study

**DOI:** 10.2196/79399

**Published:** 2026-01-20

**Authors:** Ahmed Alboksmaty, Tetiana Lunova, Ara Darzi, Ana-Luisa Neves

**Affiliations:** 1 Department of Surgery and Cancer, Institute of Global Health Innovation, Imperial College London, St Mary's Campus, Norfolk Place, London, W21PG, United Kingdom, 44 7916827620; 2 Department of Primary Care and Public Health, Imperial College London, London, United Kingdom

**Keywords:** communication, remote care, patient-physician relationship, virtual care, telehealth

## Abstract

**Background:**

The growing reliance on virtual consultations in primary care has reshaped traditional general practitioner (GP)–patient communication dynamics, presenting new challenges that affect care quality and safety.

**Objective:**

This study explores communication challenges and gaps, particularly relevant to virtual consultations compared with face-to-face interactions, as well as identifying mitigation strategies from both GPs’ and patients’ perspectives.

**Methods:**

This qualitative study employed 4 online focus group discussions with a purposive sample of UK-based GPs and patients. Data were analyzed using a deductive-inductive thematic approach with NVivo software. The extended Shannon-Weaver communication model and the Capability, Opportunity, Motivation and Behavior model guided the analysis of communication challenges and mitigation strategies, respectively. The Consolidated Criteria for Reporting Qualitative Research were followed to ensure rigorous reporting.

**Results:**

A total of 21 participants (12 patients and 9 GPs) took part in 4 online focus group discussions, 2 for patients and 2 for GPs. Six key themes on communication challenges emerged: 5 aligned with the extended Shannon-Weaver communication model (related to the sender-encoder, message, channel, receiver-decoder-feedback, and context), and a new one was inductively identified (patient autonomy and inclusivity). GPs, as senders, highlighted missing visual cues, affecting message clarity in remote communication channels. Patients, as receivers, reported difficulties explaining symptoms remotely, reduced emotional connection, and perceived empathy, linked to contextual challenges and the need for inclusive communication. Mitigation strategies were mapped to the Capability, Opportunity, Motivation and Behavior model: capability (training/resources), opportunity (triage/tools), and motivation (patient engagement/system adaptability), with participants emphasizing tailored training, standardized approaches, and flexible models to support effective and inclusive virtual communication.

**Conclusions:**

This study highlights communication gaps in virtual consultations and proposes actionable mitigation strategies. Tailored use of virtual modalities, supported by structured training and policy efforts, is essential to ensure effective and safe remote communication.

## Introduction

Primary care has become increasingly complex and demanding, driven by the variety of patients’ conditions, the multifaceted nature of health care systems, the introduction of new health technologies, and the changing needs and preferences of patients and communities [1,2]. These factors have proposed a shift toward virtual consultations, emerging a transformation in primary care delivery [3]. This trend gained considerable momentum during the COVID-19 pandemic, as telephone and video consultations became essential for maintaining health care access amid disruptions to traditional face-to-face services [3,4]. While these virtual modalities have enhanced the accessibility and sustainability of services, they also necessitate a much-needed reevaluation of the core components of communication in health care to uphold quality and safety standards [3,4].

Effective communication is fundamental to high-quality primary care, acting as the foundation for building trust, fostering mutual understanding, and delivering patient-centered care [5]. The Shannon-Weaver communication model (SWCM), a foundational framework in communication theory, offers a valuable perspective for critically analyzing GP-patient interactions across different consultation modalities (virtual and face-to-face) [6]. The extended SWCM comprises 9 components, ie, sender, encoder, message, channel, noise, decoder, receiver, feedback, and context, which collectively outline the classic communication process and can be adapted to various contexts [6].

Traditionally, the primary channel for communication between general practitioners (GPs) and patients has been face-to-face consultations. While this method facilitates direct engagement, it is not without challenges, such as time constraints, language and cultural barriers, and environmental distractions, factors that both GPs and patients have learned to navigate and challenge [7]. However, the shift to virtual consultation modalities disrupts these established dynamics, introducing new communication barriers that require tailored mitigation strategies to ensure effective and equitable care delivery [3].

Virtual consultations, whether conducted via phone or video calls, present an additional range of challenges, including technical difficulties, communication barriers, privacy and confidentiality concerns, limitations in clinical assessment, and adaptation challenges for both GPs and patients [3,8,9]. While issues with mutual understanding during in-person consultations might arise from the use of jargon or complex medical terminology, virtual consultations introduce the added difficulty of missing nonverbal cues and expressions, which can exacerbate communication gaps [10]. These distinctive challenges in remote interactions can impact the quality and safety of primary care, indicating the importance of assessing these issues in practice from the perspectives of both GPs and patients [3].

Extensive research has explored communication challenges, gaps, required skills, and improvement strategies in traditional face-to-face medical consultations within primary care [5,11,12]. However, there is limited evidence on the unique challenges and implications introduced by virtual consultation modalities. This study aims to address this gap by exploring the communication barriers and gaps specific to virtual consultations compared to traditional in-person consultations in primary care. It further seeks to identify potential strategies for mitigating these challenges based on the COM-B (capability, opportunity, motivation, and behavior) model [13], which offers a framework for identifying and presenting strategies for improvement. The assessment incorporates perspectives from both GPs and patients, who are the primary participants in the consultation process.

## Methods

### Study Design

This study adopted a qualitative approach, employing focus group discussions. The study adhered to the Consolidated Criteria for Reporting Qualitative Research (COREQ) checklist to ensure comprehensive reporting of the research processes and outcomes [14].

### Study Participants and Recruitment

Participants included patients and GPs with experience of being involved in primary care consultations, both face-to-face and remote interactions within the United Kingdom. Adult patients aged 18 years or older who could communicate in English and had lived experience of virtual consultations (video or audio) with GPs were targeted to facilitate meaningful engagement and interaction during the discussions. Patients were recruited through Valuing Our Intellectual Capital and Experience, an online platform for community engagement and involvement in research [15].

GPs were recruited through a combination of convenience and purposive sampling via the research team’s networks, followed by snowball sampling to ensure diversity among participants [16]. GP participants were required to have professional experience conducting virtual consultations (video or audio) in primary care within the UK National Health Service to ensure shared familiarity with the primary care context, including relevant policies and regulations.

All participants received, via email, a detailed participant information leaflet describing the study background, proposed methodology, and objectives. Participants were encouraged to ask questions for clarification before providing informed consent and participating in the focus groups.

### Data Collection

Four online focus groups were conducted via Microsoft Teams between June and August 2024: 2 with GPs and 2 with patients. Each group participated independently to capture distinct perspectives. The focus groups were conducted as part of a larger study aimed at identifying the safety implications of virtual consultations in primary care. A researcher (TL) with a clinical background, PhD qualification, and expertise in patient safety and digital health in primary care moderated all 4 focus groups. A senior researcher (ALN) comoderated the first GP focus group to ensure alignment of the discussions with the study’s objectives.

A semistructured topic guide was developed to facilitate interactive discussions during the focus groups, covering key aspects of communication challenges and potential solutions. The guide was piloted within the research team to ensure clarity and a logical flow. The questions in the topic guide aimed at exploring communications issues and strategies are presented in Textbox 1.

Textbox 1. **Questions relevant to discussing communication issues in the topic guide.**

- Communication problems in virtual consultations (this section aimed to explore the communication issues experienced by participants [GPs and patients] during virtual consultations).
  - What communication issues do you think can arise in virtual consultations?
  - Have you experienced any communication problems when having a virtual consultation?
  - How do you think these issues can be solved?

### Data Analysis

Microsoft Teams’ transcription feature was used to generate automatic transcripts of the discussions, which were later reviewed against the audio recordings by the focus group moderator to ensure accuracy before finalization and analysis. An interpretivist approach was adopted for this qualitative research [17], considering that participants construct meaning through their experiences and regular involvement in consultation dynamics. The analytical focus was therefore on understanding how participants interpreted and made sense of communication within virtual consultations. Transcripts were not returned to participants for further review; instead, the transcripts were directly analyzed by the research team to identify recurring themes and insights into participants’ experiences and perspectives.

**Figure 1.**
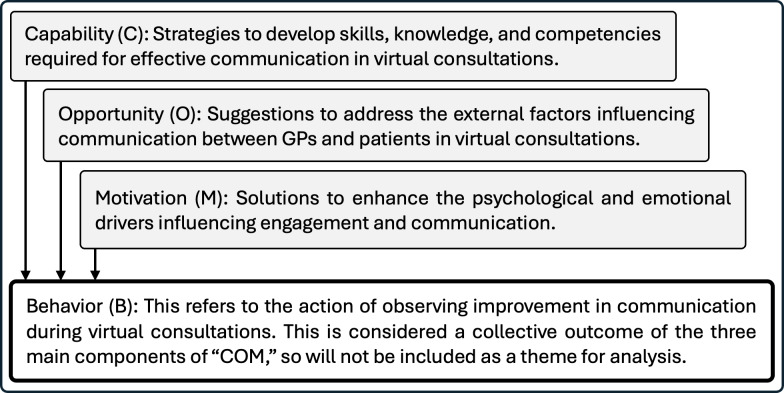
Redefined COM-B model’s components, adapted to be used for the deductive analysis of the mitigation strategies to improve communication during virtual consultations. COM-B: Capability, Opportunity, Motivation and Behavior; GP: general practitioner.

A deductive-inductive approach was employed for data analysis to ensure a comprehensive assessment while allowing for new themes to emerge [18]. The deductive analysis was guided by the components of the extended SWCM model [6], which were set as proposed themes for communication challenges and gaps. The extended SWCM originally included 9 components, which were reviewed and refined by the research team into 7 key components (Table 1), representing the major deductive themes for this analysis. For the analysis of suggested mitigation strategies, the COM-B model was adopted as the framework for deductive themes, as illustrated in Figure 1.

**Table 1.** Adapting the SWCM’s^a^ components for the deductive analysis of communication challenges and gaps.

SWCM components	Adapted definitions
Sender-Encoder	Merged definition: Combines the roles of the sender (GP^b^) and the encoder, as they are closely intertwined. The sender is the person who initiates the communication, and the encoder refers to the process of transforming thoughts into verbal and nonverbal expressions and talks. Contextualization to our research: How GPs articulate and communicate their questions, advice, and thoughts with their patients during virtual consultations.
Message	Definition: The content of the communication, including clinical advice, questions, and emotional cues. Contextualization to our research: How the clinical discussion is constructed, its clarity, and its comprehensiveness through different modalities.
Channel	Definition: The medium through which the communication is delivered, virtual (eg, video call, telephone) or face-to-face. Contextualization to our research: The limitations and merits of each modality in conveying messages effectively, particularly regarding technological or physical limitations.
Noise	Definition: Any factors that disrupt or distort the communication process. Contextualization to our research: This would include, for example, poor connectivity, background distractions, emotional and cognitive barriers.
Decoder-Receiver	Merged definition: Combines the roles of the decoder (interpreting the message) and the receiver (the person/patient for whom the message is intended). Contextualization to our research: How effectively patients understand the information, particularly the challenges posed by reduced nonverbal cues in phone consultations.
Feedback	Definition: The responses or reactions from the receiver (patient) that indicate whether the message was understood as intended. Contextualization to our research: How feedback mechanisms differ between virtual and face-to-face settings.
Context	Definition: The situational factors influencing the communication process, such as the patient-clinician relationship, and the differences in medical conditions. Contextualization to our research: How different contexts impact the communication dynamics, including the adaptability of both patients and clinicians to virtual or face-to-face settings.

a SWCM: Shannon-Weaver communication model.

b GP: general practitioner.

The initial analysis was conducted by a researcher (AA) with expertise in qualitative data analysis and the study topic. Analyses of the patients’ and GPs’ focus group datasets were first conducted independently, followed by triangulation across both sources to support a comprehensive and coherent interpretation of the findings [19,20]. This process combined a deductive approach, using the predetermined models, with an inductive approach to identify any newly emerging themes. NVivo software was used to facilitate the analysis [21].

Data adequacy was supported by the concept of information power [22], given the focused discussions, the relevance of participants recruited through purposive sampling, and the strong alignment between the data and the study objectives. During analysis, the research team observed thematic stability across the focus groups, indicating that additional data collection was unlikely to generate new insights and suggesting a level of data saturation appropriate for the study aims [23]. Preliminary themes were further refined through iterative analysis and discussions among all members of the research team to reach a consensus on the final themes.

### Ethical Considerations

Ethics approval for this study was obtained from the Imperial College Research Ethics Committee (ICREC 6833567) before commencing the recruitment and study processes. All participants provided informed consent prior to taking part in the focus group discussions. This consent was obtained after participants had reviewed comprehensive participant information leaflets detailing the study’s aims and procedures, and after they had the opportunity to ask questions and receive clarification on any aspect of the study. To protect privacy and confidentiality, data were pseudoanonymized; identifiable details were removed after verification of transcription accuracy, and only deidentified qualitative data were used in the analysis. Participants received a £30 (US $40.36) voucher as a token of appreciation for their time and contribution.

## Results

### Participant Characteristics

A total of 21 participants (12 patients and 9 GPs) were involved in 4 focus group discussions; 2 patient groups, each with 6 participants, and 2 GP groups with 5 and 6 participants, respectively. None of the invited participants refused to take part. Each online focus group lasted for an average duration of 90 minutes. All participating GPs (n=9) were based in England, along with the majority of patients (10/12). The participating GPs had a mean of 5.33 (SD 4.06) years of National Health Service experience, with a range spanning from 2 to 16 years. Table 2 provides an overview of the participants’ characteristics.

**Table 2.** Self-reported participant characteristics.

Characteristic	Patients	GPs^a^
Total number of participants, n (%)	12 (57.14)	9 (42.86)
Age bands, n (%)		
18-‐29 years (n=1, 4.76%)	1 (4.76)	0 (0)
30-‐39 years (n=10, 47.62%)	3 (14.29)	7 (33.33)
40-‐49 years (n=4, 19.05%)	2 (9.52)	2 (9.52)
50-‐59 years (n=2, 9.52%)	2 (9.52)	0 (0)
≥60 years (n=4, 19.05%)	4 (19.05)	0 (0)
Missing (n=0, 0%)	0 (0)	0 (0)
Sex, n (%)		
Female (n=13, 61.90%)	8 (38.10)	5 (23.81)
Male (n=8, 38.10%)	4 (19.05)	4 (19.05)
Missing (n=0, 0%)	0 (0)	0 (0)
Geographic location, n (%)		
Bournemouth (n=1, 4.76%)	1 (4.76)	0 (0)
Bristol (n=1, 4.76%)	1 (4.76)	0 (0)
Cambridge (n=2, 9.52%)	0 (0)	2 (9.52)
Edinburgh (n=1, 4.76%)	1 (4.76)	0 (0)
Leicester (n=1, 4.76%)	0 (0)	1 (4.76)
Lisburn (n=1, 4.76%)	1 (4.76)	0 (0)
London (n=9, 42.86%)	3 (14.29)	6 (28.57)
Newcastle Upon Tyne (n=4, 19.05%)	4 (19.05)	0 (0)
Walsingham (n=1, 4.76%)	1 (4.76)	0 (0)
Missing (n=0, 0%)	0 (0)	0 (0)
Ethnic group, n (%)		
Arab (n=2, 9.52%)	1 (4.76)	1 (4.76)
Asian or Asian British (n=6, 28.57%)	2 (9.52)	4 (19.05)
Black or Black British (n=2, 9.52%)	2 (9.52)	0 (0)
Mixed (n=1, 4.76%)	1 (4.76)	0 (0)
Other (n=1, 4.76%)	0 (0)	1 (4.76)
White (n=9, 42.87%)	6 (28.57)	3 (14.29)
Missing (n=0, 0%)	0 (0)	0 (0)

a GP: general practitioner.

### Communication Challenges and Gaps

#### Overview

Suggestions from GPs and patients were overall aligned, reflecting shared struggles in communication during virtual consultations and a common desire for improvement. Six themes were identified through the data analysis of both patient and GP focus groups, comprising 5 deductive themes derived from merging some of the SWCM components and 1 inductive theme [6]. The 5 deductive themes included communication challenges related to (1) sender-coder, (2) message, (3) channel, (4) receiver-decoder-feedback, and (5) context with modality appropriateness. A newly identified inductive theme was labeled patient autonomy and inclusivity. A description of each theme and its subthemes is provided in Table 3.

**Table 3.** Major themes, description, and subthemes identified through the data analysis.

Major theme	Description	Subthemes
Theme 1: Sender–Encoder	How GPs^a^ articulate and communicate their questions, advice, and thoughts with their patients during virtual consultations.	Issues in continuity and coordination of care Misunderstanding, bias, and missing clinically important points Lack of empathy and emotional engagement
Theme 2: Message	How the clinical discussion is constructed, its clarity, and its comprehensiveness through different modalities.	Less clarity and comprehensiveness in virtual consultations Limited diagnostic visualization during phone consultations
Theme 3: Channel	The limitations and merits of each consultation modality in conveying messages effectively, particularly regarding technological or physical limitations.	Technical and organizational barriers Physical barriers in virtual communication Accessibility and reliability issues for stable connection in virtual consultations
Theme 4: Receiver–Decoder–Feedback	How patients interpret and understand clinical information and discussions and how their responses and interactions indicate understanding and engagement.	Lack of trust and confidence in virtual consultations Communication issues that affect understanding, including language barriers Resistance by patients to engage in virtual consultations Unclear understanding and missing engagement cues Missing natural interactions and non-verbal cues in phone consultations
Theme 5: Context and modality appropriateness	The situational factors and suitability of communication methods that influence the effectiveness and engagement of interactions between GPs and patients.	System and structural limitations Influence of cultural and social norms Questionable patient expectations and trust No standardized system to decide on the consultation type
Theme 6: Patient autonomy and inclusivity	How patient autonomy and inclusivity influence communication between GPs and patients, focusing on the extent to which patients can control their consultation choices and the barriers to full participation in virtual care.	Barriers to choosing consultation modality Technical challenges faced by some patient groups in engaging with virtual consultations Patient preferences for in-person consultations Inequality in access to technology

a GP: general practitioner.

#### Theme 1: Sender–Encoder (GPs)

Virtual consultations occasionally disrupted clinicians’ and patients’ ability to interpret emotional and clinical cues, leading to a mutual sense of disconnection that affects relational quality. GPs expressed concerns about missing nonverbal and visual clinical signs during phone consultations, negatively affecting diagnostic decision-making. They also highlighted the extra efforts needed for maintaining engaging conversations with patients remotely, a difficulty further exacerbated by increasing workload pressures. Patients, in turn, reported feeling a lack of empathy and emotional connection during virtual consultations, highlighting a perceived disconnect between themselves and their GPs in virtual settings, particularly in phone consultations.

*Missing non-verbal communication, the stuff we wouldn't get in a virtual [phone] consultation, you can't see them [patients] walk into the room, can't see how they're sitting, or notice the little things that might make you think differently.*

[GP 3.3]

*Many of the emotions and empathy, at least towards my case, are almost completely gone when it comes to phone and video consultations*.

[Patient 2.1]

#### Theme 2: Message (Content of the Medical Consultation)

The lack of direct interaction in virtual consultations complicated symptom description and clinical explanation, making it harder for both patients and GPs to achieve clarity, especially when addressing complex or sensitive issues. Patients raised significant concerns about the difficulty of clearly describing symptoms and medical complaints over the phone. Likewise, GPs reported challenges in effectively explaining diagnostic reasoning and management plans to patients and ensuring their understanding during virtual consultations, which is of more concern during phone calls. These issues became particularly challenging when discussing sensitive or complex medical conditions.

*…Because the doctor can't pick up on non-verbal cues that might explain a complaint or tell if the patient wants to say something else.*

[Patient 3.2]

#### Theme 3: Channel

Participants reflected that the risk of technical unreliability in remote communication tools essentially reduces the perceived quality of virtual consultations. Patients expressed increased concerns about the timing of their appointments, often disrupted by delays in calls, the reliability of their internet or phone connections, and the challenge of effectively describing symptoms over the phone, particularly for non-native speakers. Similarly, GPs reported concerns about dropped calls or lost connections during consultations, as well as issues with the quality of sound and images shared online, which could hinder effective communication.

*The number of times I've called patients—too many—they’ve had a really bad signal, no alternative number to call, or they’re out in a supermarket or somewhere else, making me just can’t understand a word they’re saying.*

[GP 4.3]

#### Theme 4: Receiver–Decoder–Feedback

Persistent challenges in trust, confidence, and equitable access to care influenced patient engagement with virtual consultations, with the absence of nonverbal interactions over the phone often weakening the mutual understanding essential for safe and reassuring care. Despite the normalization of virtual consultations for GP appointments in the United Kingdom, particularly since the COVID-19 pandemic, patients continued to express a lack of confidence and trust in this method for addressing serious medical conditions. This has been perceived by GPs as a form of resistance, especially among elderly patients, toward accepting virtual consultations as the only method of communication, even when deemed clinically safe.

*There’s a lot of reluctance towards virtual consultations across the country… some people say there’s no place for virtual consultations because they’ve been brought up with the face-to-face discussion model.*

[GP 1.4]

Patients further expressed concerns about equity in accessing and benefiting from remote care, particularly among individuals with special needs, such as those with hearing impairments, mental health issues, in need of an interpreter, or learning difficulties. Both GPs and patients reported ongoing challenges with interactive feedback during virtual consultations, largely due to the reliance on verbal cues alone, with potentially facial reactions in video consultations, to convey messages and management plans on most occasions.

#### Theme 5: Context and Modality Appropriateness

Both GPs and patients emphasized that effective communication depends on aligning consultation modality with clinical needs and contextual expectations. GPs highlighted the need for a robust triage system and effective coordination policies to ensure patients receive the most appropriate and convenient consultation modality tailored to their individual needs. Patients echoed this sentiment, emphasizing that communication during medical appointments extends beyond the consultation itself and is influenced by health care system structure, social norms, and community culture. Bridging these perspectives, both groups stressed that aligning the consultation modality with patient expectations and needs is crucial for building trust and ensuring effective communication.

*I needed to be present with the doctor. I needed to be there, face-to-face, when talking about sensitive and difficult things. I think sometimes GPs need to acknowledge that a quick call or video call isn't the right way to talk to a patient at certain times.*

[Patient 1.1]

#### Theme 6: Patient Autonomy and Inclusivity

Patients shared a wish to have their voices heard beyond their clinical symptoms, particularly in deciding the type of consultation they receive with their GP. They further explained that some patients struggle to engage, express their emotions, or maintain focus during remote conversations, making in-person appointments more suitable for their personal traits, regardless of their symptoms. Conversely, patients with demanding jobs or caregiving responsibilities may prefer communicating remotely for their convenience. GPs acknowledged this perspective, emphasizing that person-centered and equitable care requires understanding each patient’s preferences and capabilities while also assessing their medical needs.

*I would like a bit more patient choice over which consultations you may have. There may be some conditions where you're quite happy to talk about them over the phone, while with others, you know you’d be better off having an in-person or video consultation.*

[Patient 3.2]

### Mitigation Strategies to Improve Communication

The proposed mitigation strategies targeted the identified challenges and gaps, which were categorized into 3 deductive themes (capability, opportunity, and motivation) based on the COM-B model [13]. Table 4 outlines these major themes and the associated subthemes based on the data analysis.

**Table 4.** Theme of the mitigation strategies based on the capability, opportunity, motivation, and behavior model.

Major theme	Subthemes
Theme 1: Capability	Training and skill development for virtual consultations Resource provision and standardization Enhancing patient understanding
Theme 2: Opportunity	Triage and risk mitigation Enhancing communication through supplementary materials Addressing barriers to effective communication
Theme 3: Motivation	Patient and doctor preferences in consultation modalities Supporting patient preparedness and engagement Monitoring and adapting models of care

#### Theme 1: Capability

Training and skill development for virtual communication was highlighted by both patients and GPs as essential for improving interactions during virtual consultations. It was noted that strong communication skills in face-to-face settings do not necessarily translate to effective communication over the phone, and vice versa. Therefore, it was suggested that medical students and doctors in training should gain practical experience in conducting virtual consultations and leading virtual clinical appointments. Additionally, there was a shared call for equipping GPs with modern tools and reliable infrastructure to enable seamless and effective communication with patients remotely.

*I think we need to train our GP trainees using a different model of communication.*

[GP 1.4]

*…training [of GPs] on using specific platforms and building rapport over the phone to aid patient-doctor communication, I think, is important, how you do that over the phone to achieve the maximal effect.*

[Patient 3.2]

#### Theme 2: Opportunity

The primary recommendation in this theme was to set a standardized triage system and clear protocols to determine the most suitable consultation modality based on each patient’s individual needs, preferences, and characteristics. Offering video consultations as a standard option, in addition to phone appointments, was also suggested as a way to enhance engagement and build stronger professional doctor-patient relationships. Another proposal involved maintaining a registry of patients who face barriers to virtual consultations, such as those living in areas with poor signal, experiencing language barriers, suffering from hearing impairments, or having complex medical conditions.

*In our practices, there are two completely separate approaches. One practice has a policy that if patients want virtual, they get virtual, and if they want face-to-face, they get face-to-face. In the other practice, the receptionist decides.*

[GP 3.4]

#### Theme 3: Motivation

A GP’s comfort and familiarity with different consultation modalities were identified as important factors influencing communication quality, whether virtually or face-to-face. Some participants proposed a system that allocates patients to GPs based on their preferred consultation modality, for instance, assigning virtual consultations to GPs who favor them, while those who prefer direct communication handle face-to-face appointments. However, concerns were raised about the potential impact of this approach on continuity of care.

*One approach could be to ask patients whether they would prefer a virtual consultation with a new doctor or one they are familiar with. Personally, I would feel more comfortable talking to a doctor I know in person.*

[GP 2.4]

Patients also suggested offering incentives to encourage engagement in virtual consultations, such as implementing a fast-track appointment booking system for those opting for virtual appointments. However, some participants noted potential inequality issues with such incentives, as certain patients may be unable to choose virtual consultations due to personal or medical needs. Additionally, a system to promptly update patients about any changes to their appointment times was recommended to help them prepare accordingly and feel more confident in the system.

## Discussion

### Summary of Results

This study adopted a qualitative approach using focus groups with GPs and patients to identify communication challenges in primary care virtual consultations compared to traditional face-to-face appointments, as well as exploring mitigation strategies. Four online focus groups were conducted, 2 with GPs (n=9) and 2 with patients (n=12).

Six major themes emerged regarding the challenges and gaps in virtual consultations, 5 of which align with the SWCM model: sender-encoder, message, channel, receiver-decoder-feedback, and context with modality appropriateness. An additional theme, patient autonomy and inclusivity, was identified inductively. For GPs as sender-encoders, misinterpretations and missed clinically significant cues were major concerns. The “message” was often compromised by limited clarity in dialog and difficulties in communicating diagnostic information virtually. Technical issues, poor connectivity, and accessibility barriers complicated the remote “channels” of communication. Patients, as receiver-decoders, reported a lack of trust in discussing complex medical needs remotely and perceived reduced empathy and emotional engagement from GPs in virtual consultations. Social norms and cultural factors, especially among elderly patients, shaped preferences and communication styles, highlighting the importance of “context and modality appropriateness.” Furthermore, patient autonomy and inclusivity in choosing virtual consultations influenced their engagement and willingness to communicate effectively during virtual primary care appointments.

Proposed mitigation strategies were mapped to the COM-B model: capability (training and resource provision), opportunity (triage systems and supplementary communication tools), and motivation (patient engagement and system adaptability). Participants highlighted the need for tailored capacity building through virtual-communication-focused training and skills development, supported by resource provision and standardized approaches. Recommendations to enhance the opportunity for better communication in virtual consultations included implementing triage and risk mitigation strategies, using supplementary materials to support remote interactions, and continuously identifying and addressing communication barriers. To strengthen motivation among both GPs and patients, participants emphasized the importance of respecting individual preferences for consultation modality, offering preparatory guidance and materials to support patient engagement, and ensuring flexibility and adaptability in care models.

### Findings in Context of Existing Literature

Consistent with our findings, previous studies have identified common challenges in virtual consultations. Patients often struggle to articulate complex symptoms over the phone, while GPs face difficulties ensuring patient understanding of medical information and management plans [24]. These challenges are particularly pronounced in sensitive discussions, cases involving complex health needs, conditions requiring physical examination [25], and with new patients unfamiliar to the GP [26,27]. Some conditions, such as mental health concerns, raise questions about the appropriateness of virtual consultations on some occasions [28]. Our participants described a perceived lack of empathy in virtual appointments, contributing to dissatisfaction in mental health care. However, previous studies reported contrasting evidence, suggesting that offering timely virtual appointments may enhance mental health care by enhancing accessibility to medical advice whenever needed [3,28,29]. This inconsistency in evidence for some medical conditions highlights the importance of contextualizing and adapting care and communication models, including the choice of consultation modality, to align with individual patient needs and clinical circumstances [11]. It is also important to note that patient preferences for communicating remotely versus face-to-face are influenced not only by their health needs but also by personal characteristics [30]. Evidence shows that younger individuals, those with busy schedules, and highly educated patients tend to favor virtual appointments for convenience and accessibility [3,31].

Existing literature has tended to identify similar communication challenges in primary care consultations, but these are often presented in a fragmented manner [26,29,32]. Our study synthesizes these challenges within a structured, theoretically informed framework [6], with particular emphasis on virtual consultations as an emerging norm in primary care [3]. By framing communication issues as a shared responsibility between patients and GPs [33], we offer a more nuanced interpretation that supports practical approaches to mitigation. Through the application of the adapted SWCM framework [6], we further highlight how patient, clinician, technological, and contextual factors intersect, enabling us to propose targeted interventions that may lead to meaningful and sustained improvements in communication practices.

Importantly, communication challenges in virtual consultations extend beyond the consultation itself, involving both pre- and post-encounter interactions [32,34,35]. A qualitative study conducted in Australia highlighted pre-consultation patient-related factors that influence GP-patient communication, particularly health literacy and familiarity with digital platforms [26]. Many patients struggle with completing pre-consultation forms required for describing their symptoms and concerns for booking virtual appointments, especially when selecting appropriate terms to describe their symptoms [32]. From a GP’s perspective, familiarity with the patient, as well as access to and time for reviewing medical records, plays a crucial role in shaping communication style and ensuring a high-quality virtual consultation [27,36]. Existing tools also provide structured checklists and guidance to support high-quality virtual consultations, with communication highlighted as a central component [37]. For example, the Telehealth Etiquette Competency Checklist (TECC) emphasizes communication alongside technological, environmental, and confidentiality considerations [37].

Effective communication remains essential even after the consultation itself, particularly in follow-up messaging, referrals, and sharing components of the agreed-upon management plan [34]. The widespread adoption of virtual consultations has raised concerns regarding continuity of care, as it may challenge the foundation of building a strong professional relationship between GPs and their patients, which is an essential factor in enhancing the consultation experience and overall quality of care [38]. Ensuring clarity in follow-up plans after virtual consultations is crucial, particularly in guiding patients on how to seek further care if their concerns persist or if the initial virtual visit does not fully address their health needs [8,35]. Clear and efficient remote communication can help reduce unnecessary follow-up visits, whether in primary care or to hospital and ambulatory care [3].

To mitigate the identified communication barriers and enhance interactions during virtual consultations, GPs often use verbal affirmations, such as “yes” and “I see,” to prove active listening [33]. They may also verbally narrate any concurrent tasks, such as reviewing investigation results or previous reports, to maintain transparency with patients for reassurance. Such capacity-building requirements for these supporting skills have been proposed by the participants in our study to improve communication.

Evidence suggests that, when feasible, video consultations may be preferable for initial appointments to establish rapport, for assessments requiring observation of physical signs [25], and for counseling appointments. Existing literature also offers evidence-based guidelines, emphasizing aspects regarding the knowledge, skills, attitudes, and teaching strategies required for high-quality videoconferencing, which primary care teams can adapt to their local context [39]. However, no clear advantage or superiority of video consultations over face-to-face interactions has been established [40]. The collective evidence from broader literature indicates that both GPs and patients generally prefer communication through face-to-face consultations, followed by video calls when feasible, with phone consultations being the least preferred [41]. However, this hierarchy may vary depending on the context; for instance, when the consultation is for routine medication renewal, or patients live far away from their GP surgeries, phone or video consultations could be more practical [42].

### Strengths and Limitations

This study focuses on the core aspect of virtual consultation -communication- as a critical determinant of both satisfaction and patient preference in clinical practice [5]. Focus groups were employed due to their ability to foster an in-depth dialog among participants and to identify shared concerns effectively [43,44]. However, focus groups could potentially allow vocal participants to dominate the discussion and the possibility that some individuals may share less in-depth reflections in a group setting [45]. To mitigate these risks, the moderator had substantial experience in conducting similar research, ensuring balanced participation and providing sufficient time for individuals to share their views [45]. Additionally, in terms of methodology, future research using direct observation or other objective methods could reveal communication nuances that focus group discussions may miss [46]. This would enable a clearer assessment of communication aspects in different consultation modalities.

The analysis was strengthened by the use of 2 theoretical frameworks to structure the data analysis and guide its interpretation: the SWCM framework [6], which addresses the challenges and gaps, and the COM-B model [13], which informs the development of mitigation strategies. Our analysis integrated insights from both GP and patient transcripts, emphasizing that effective communication is inherently reciprocal and requires active engagement from both parties.

While we aimed to recruit a diverse patient population, further research is needed to explore communication challenges among particular groups with potential distinct needs, such as patients requiring interpreters or those with mental health conditions and complex chronic illnesses like cancer. Additionally, all participating GPs were based in England, with an average of 5.4 years of experience. Future studies should include GPs at varying career stages and across different practice settings to identify role-specific communication challenges and potential training needs. Moreover, our study categorized both video and telephone consultations under the broad term of virtual consultations; however, each modality presents unique communication dynamics that warrant further investigation.

### Implications for Research, Policy, and Practice

Virtual consultations have become an integral part of the “norm” in primary care, whether in the UK or globally. Therefore, it is essential to optimize remote communication to uphold patient safety and care quality [3]. The communication challenges and mitigation strategies identified in this study, structured using theoretical frameworks, provide actionable themes to be considered by policymakers and practitioners for improvement plans. For example, targeted communication training for GPs, ensuring that GP practices are equipped with stable and reliable technology for virtual consultations, and implementing guidelines to allocate virtual appointments to patients who are both medically and personally suited for them are all key areas for intervention.

Further research should focus specifically on GP-patient communication during virtual consultations, examining its impact on patient satisfaction and clinical outcomes, including safety. Policy efforts should extend beyond the provision of technology and infrastructure to include structured training programs that equip GPs and other clinicians with the skills necessary for effective remote communication. The training efforts should target not only qualified GPs but also undergraduate medical students and other healthcare professionals who are involved in patient care provision, potentially through virtual appointments [12]. In practice, the allocation of virtual consultations should consider both the GP’s level of experience in remote communication and the patient’s health condition and background. Communication in healthcare should be viewed holistically, not merely as spoken language, but as the means through which care is delivered, tailored to the needs of each patient.

### Conclusion

This study highlights the pivotal role of communication in virtual consultations, highlighting existing gaps and potential strategies for improvement in primary care. Through focus groups with GPs and patients, we identified key communication challenges and potential mitigation strategies. While virtual consultations offer convenience, their use should be tailored to individual patient needs and clinical contexts. Future research should explore the distinct communication dynamics of different virtual modalities in primary care, informing policymakers and practitioners with avenues for improvement to ensure equitable, high-quality, and patient-centered primary care delivery.

## Abbreviations

COM-B: Capability, Opportunity, Motivation and Behavior model
COREQ: Consolidated Criteria for Reporting Qualitative Research
GP: general practitioner
SWCM: Shannon-Weaver communication model
